# Defining the pH* Scale in Methanol: Determination of Accurate Values for the Solvation Free Energies of CH_3_OH_2_^+^ and CH_3_O^–^ in Methanol

**DOI:** 10.1021/acs.jpca.5c03979

**Published:** 2025-10-25

**Authors:** Antonio R. Cunha, José M. Riveros, Sylvio Canuto, Kaline Coutinho

**Affiliations:** † 37892 Universidade Federal do Maranhão, UFMA, Campus Balsas, Balsas CEP 65800-000, Maranhão, Brazil; ‡ Instituto de Química, Universidade de São Paulo, Cidade Universitária, São Paulo CEP 05508-000, Brazil; § Instituto de Física, Universidade de São Paulo, Cidade Universitária, São Paulo CEP 05508-090, Brazil

## Abstract

A correction factor for the autoprotolysis constant of methanol is proposed in the present work to obtain thermodynamic data for the standard solvation free energies of CH_3_OH_2_^+^ and CH_3_O^–^ ions in methanol and p*K*_a_^*^. Using this corrected constant, *K*_MOH_^*^, along with known values for the standard solvation free energy of proton Δ*G*_sol_^*^(H^+^) and of the methanol molecule Δ*G*_sol_^*^(CH_3_OH), in its own liquid, in three different thermodynamic cycles, we obtain Δ*G*_sol_^*^(CH_3_OH_2_^+^) = −91.41 ± 2.76 kcal mol^–1^, Δ*G*_sol_^*^(CH_3_O^–^) = −88.36 ± 2.10 kcal mol^–1^, and p*K*_a_^*^(methanol) = 22.67 ± 2.97. To validate our approach, we applied the same thermodynamic cycles for water in its own liquid, resulting in experimental values of Δ*G*_sol_^*^(H_3_O^+^) = −110.20 ± 1.91 kcal mol^–1^, Δ*G*_sol_^*^(OH^–^) = −104.60 ± 0.25 kcal mol^–1^, and p*K*_a_(water) = 15.73 ± 1.42. Employing quantum mechanics calculations combined with Monte Carlo simulation, we calculated the standard deprotonation free energy and the p*K*_a_ values of water and methanol in their respective liquids. These calculations were performed using an explicit model of the solvent, with Free Energy Perturbation theory in Monte Carlo simulation (FEP-MC), three different pure implicit solvation models (HF-PCM, the Conductor-like Polarizable Continuum Model (C-PCM), the solvation model based on density (SMD)), and a hybrid model (cluster-SMD). Excellent agreement with experimental data was achieved using FEP-MC, HF-PCM, and cluster-SMD. Methanol is the simplest alcohol, and its p*K*_a_^*^ value is a critical parameter in chemical and biological systems. Hence, its understanding, along with its pH* scale, enables better control and utilization of methanol in diverse applications, ranging from pharmaceuticals to industrial processes.

## Graphical abstract

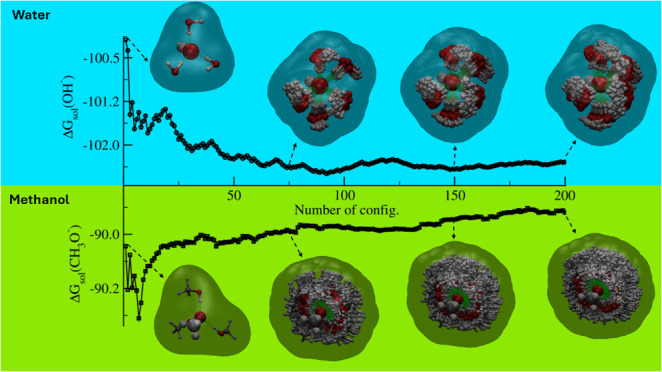

## 1 Introduction

The autoprotolysis constant *K*_ap_ of a solvent is an important and fundamental property for determining the “normal pH scale.” ^1,2^ In water, the autoprotolysis constant is defined as *K*_ap_ = *K*_W_ = [H^+^]­[OH^–^] or *K*_W_ = [H_3_O^+^]­[OH^–^] because the naked proton (H^+^) does not exist in liquid water. The value of this constant for water at 25 °C is well-known, *K*_W_ = 10^–14^ (p*K*_W_ = −log­(*K*_W_) = 14). It is used to define the pH scale in aqueous solutions^3^ and to obtain important chemical properties in aqueous solution,^4–7^ such as the neutral solution $\mathrm{pH}=\frac{pK_{W}}{2}$ = 7.0 where [H^+^] = [OH^–^] or [H_3_O^+^] = [OH^–^], and the p*K*_a_ = p*K*_W_ + log­[H_2_O] = 15.7 where [H_2_O] = 55.5 mol L^–1^. For nonaqueous solvents, there has been intense activity directed toward the acquisition of data for media that are of interest to chemistry and chemical engineering. As a result, autoprotolysis constants have been determined for several solvents.^8–25^ Although these constants have been known for some time, a pH scale for these solvents has yet to be defined, and pH measurements in nonaqueous media remain a challenging problem. Various IUPAC reports have emphasized the importance of these constants for chemistry in nonaqueous solvents, and efforts have been made to adopt criteria for the standardization of pH measurements in nonaqueous solvents and in aqueous–organic solvent mixtures.^26–28^

Over the years, a number of publications have reported measurements made in nonaqueous solvents with pH meters calibrated with specific buffer solutions.^1,26,29–32^ The acidity constant values, p*K*_a_^*^, were thus determined for many organic compounds. The asterisk indicates that these values refer to measurements relative to an ideal dilute solution in the same solvent. Although such p*K*_a_^*^ values are known, there is no clear thermodynamic significance attached to these constants.^1,29^ In fact, different pH* scales for different solvents have been developed based on the values of specific buffers, with pH* measurements based on a pH meter standardized with appropriate pH* buffers, and with the acidity constant for a compound (p*K*_a_^*^) determined from the pH* reading. Alternatively, when a pH meter standardized with aqueous pH buffers is used and the appropriate correction factor δ value is known, the pH* is determined using a simple relationship between pH* and the pH_app_ (the pH meter reading, also called pH apparent): pH* = pH_app_ + δ.^1,29^

A nonaqueous solvent that has received considerable attention in this field is methanol. This is a solvent of great importance and very common in organic chemistry because several compounds of industrial chemistry interest are soluble in methanol. The autoprotolysis constant of methanol at *T* = 25 °C is known to be *K*_ap_ = *K*_MOH_ = 10^–16.7^,^20,23^ determined from the apparent ionic product in water–methanol mixtures. Some early work, primarily those by Bates^1^ and by De Ligny and coauthors,^33,34^ was carried out with the aim of defining a pH* scale for this solvent. For example, De Ligny et al.^33,34^ have shown that the difference between the pH* and pH_app_ scale in methanol is δ = 2.34. More recently, Beckers and Ackermans^35^ used capillary zone electrophoresis to report a value of δ = 2.25 for methanol, in good agreement with the previous value. For the purpose of the present work, we can consider an average value of δ = 2.30. Thus, in order to introduce a clear thermodynamic significance to *K*_ap_^*^ and p*K*_a_^*^ for nonaqueous solvent, a correction factor is needed for the original value of the autoprotolysis constant of methanol, namely, *K*_MOH_^*^ = 10^–2δ^ *K*_MOH_ = 10^–(2δ+16.7)^, where *K*_MOH_ = [H^+^]­[CH_3_O^–^] = 10^–16.7^ (or *K*_MOH_ = [CH_3_OH_2_^+^]­[CH_3_O^–^] = 10^–16.7^, considering that the naked proton H^+^ does not exist also in liquid methanol). A neutral solution of methanol would then yield a $pH_{\mathrm{app}}=\frac{pK_{\mathrm{MOH}}}{2}=$ 8.35 and a p*K*_a_ = p*K*_MOH_ + log­[CH_3_OH] = 18.1, where [CH_3_OH] = 24.5 mol L^–1^. Using the relationship proposed previously,^1,29^ pH* = pH_app_ + δ (where δ = 2.30 for methanol), a methanol neutral solution has a pH* = 10.7. This corrected value for the autoprotolysis constant of methanol, *K*_MOH_^*^ = 10^–2δ^ *K*_MOH_ = 10^–21.3^, then leads to a p*K*_a_^*^ = 22.7 in the methanol scale. Figure 1 shows an illustration of both scales: pH (for water) and pH* (for methanol).

**Figure 1:**
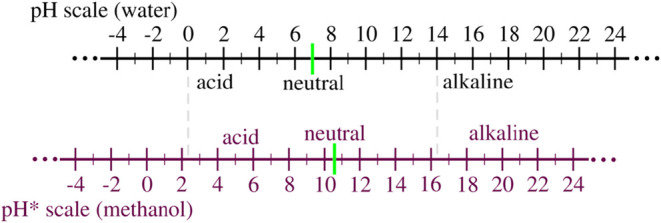
An illustration of the pH scale for water and pH* = pH_app_ + δ scale for methanol, where δ = 2.30. The values of the neutral solution are pH = 7.0 in water and pH* = 10.7 in methanol, and a p*K*_a_ = 15.7 in water and a p*K*_a_^*^ = 22.7 in methanol.

In this work, the value of the corrected autoprotolysis constant of methanol *K*_MOH_^*^ is used to determine the experimental values of the solvation free energies of the methoxonium, Δ*G*_MOH_^*^(CH_3_OH_2_^+^) and the methoxide, Δ*G*_MOH_^*^(CH_3_O^–^), ions in methanol solution, where the G* symbol indicates the free energies referenced to the 1 mol L^–1^ standard state.^36–38^ To the best of our knowledge, no previous values have been reported for the solvation free energies of the methoxonium and the methoxide ions in methanol solution.

Thermodynamic cycles from three common processes can be used to obtain the values of the experimental free energies, i.e., (1) SH → S^–^ + H^+^, where SH is the protic solvent molecule of interest, S^–^ is the deprotonated form of SH, and H^+^ is the naked proton; (2) 2SH → S^–^ + SH_2_^+^, where SH_2_^+^ is the protonated form of SH; and (3) SH_2_^+^ → SH + H^+^. Similar thermodynamic cycles have been previously used^39–42^ to identify the solution acidity, Δ*G*_sol_^(1)^, from process (1) and the solution basicity, Δ*G*_sol_^(3)^, from process (3), while process (2) is a combination of process (1) and (3). The values of Δ*G*_sol_^*^(SH_2_^+^) and Δ*G*_sol_^*^(S^–^) can then be obtained by using relationships deduced from these thermodynamic cycles. To validate this approach, we have applied the same thermodynamic cycles for water solution and identified the experimental values of the solvation free energies of the hydronium, Δ*G*_W_^*^(H_3_O^+^), and the hydroxide, Δ*G*_W_^*^(OH^–^), ions in water. Furthermore, we compare the values obtained in this work with the most reliable available experimental results for the hydronium^43^ and for the hydroxide^36–38,41–44^ ions.

For comparative purposes, we have also carried out a theoretical study to calculate the values of Δ*G*_W_^*^(OH^–^) and Δ*G*_W_^*^(H_3_O^+^) in water, and Δ*G*_MOH_^*^(CH_3_O^–^) and Δ*G*_MOH_^*^(CH_3_OH_2_^+^) in methanol. The theoretical approach was based on various models of solvation, including an explicit solvent model,^45^ three different pure implicit solvent models,^46–49^ and a hybrid model,^50,51^ i.e., with the solute in the presence of implicit and explicit solvent molecules.

## 2 Determination of Experimental Data for Δ*G*_sol_^*^(S^–^) and Δ*G*_sol_^*^(SH_2_^+^)

### 2.1 Methodology

The procedure to estimate the experimental solvation free energies of OH^–^ and H_3_O^+^ ions in aqueous solution and of CH_3_O^–^ and CH_3_OH_2_^+^ ions in methanol solution was based on the three thermodynamic cycles shown in Schemes 1–3. These thermodynamic cycles combine the protonation–deprotonation process of the solvent molecule in the gas phase and in solution. Scheme 1 considers the dissociation process of the neutral solvent molecule, SH, into deprotonated species, S^–^, and proton, H^+^. Scheme 2 considers the dissociation process of SH into the deprotonated species, S^–^, and the protonated solvent molecule, SH_2_^+^. Meanwhile, Scheme 3 considers the dissociation process of SH_2_^+^ into SH and the proton H^+^.

**Scheme 1:**
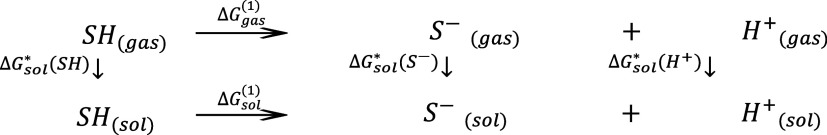
Thermodynamic Cycle 1 for the Acidity Reaction Involving the Direct Dissociation of the Neutral Species SH into the Anionic Species S^–^ and a Proton H^+^ in the Gas Phase and in Solution^a^

a Δ*G*_gas_^(1)^ and Δ*G*_sol_^(1)^ are the gas phase and solution acidity, respectively. For the methanol case, SH = CH_3_OH, S^–^ = CH_3_O^–^, and sol = MOH, and for water, SH = H_2_O, S^–^ = OH^–^, and sol = W.

**Scheme 2:**
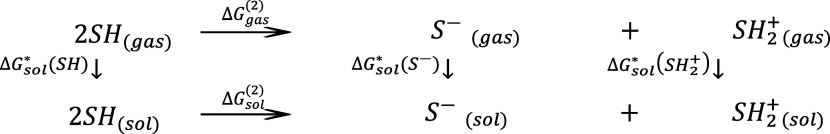
Thermodynamic Cycle 2 for the Heterolytic Dissociation of the Neutral Species SH into the Anionic Species S^–^ and the Cationic Species SH_2_^+^ in the Gas Phase and in Solution^a^

a Δ*G*_gas_^(2)^ and Δ*G*_sol_^(2)^ are the gas phase and solution free energies of the acid–base reaction, respectively. For the methanol case, SH = CH_3_OH, S^–^ = CH_3_O^–^, SH_2_^+^ = CH_3_OH_2_^+^, and sol = MOH, and for water, SH = H_2_O, S^–^ = OH^–^, SH_2_^+^ = H_3_O^+^, and sol = W.

**Scheme 3:**
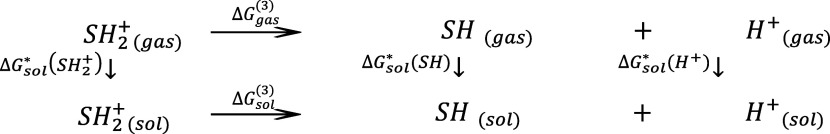
Thermodynamic Cycle 3 of the Basicity Reaction Involves the Direct Dissociation of the Cationic Species SH_2_^+^ into the Neutral Species SH and the Proton H^+^ in the Gas Phase and in Solution^a^

a Δ*G*_gas_^(3)^ and Δ*G*_sol_^(3)^ are the gas phase and solution basicities, respectively. For the methanol case, SH_2_^+^ = CH_3_OH_2_^+^, SH = CH_3_OH, and sol = MOH, and for water, SH_2_^+^ = H_3_O^+^, SH = H_2_O, and sol = W.

The thermodynamic cycle shown in Scheme 1 can be used to obtain the experimental value of Δ*G*_sol_^*^(S^–^), using the gas phase and solution acidity of the solvent and the corresponding solvation free energies of the species, as shown in eq 1

1 $$\Delta G_{sol}^{*}\left(S^{-}\right)=-\Delta G_{gas}^{\left(1\right)}+\Delta G_{sol}^{*}\left(SH\right)-\Delta G_{sol}^{*}\left(H^{+}\right)+\Delta G_{sol}^{\left(1\right)}$$

where Δ*G*_gas_^(1)^ is the gas phase acidity, Δ*G*_sol_^(1)^ is the solution acidity, Δ*G*_sol_^*^(SH) and Δ*G*_sol_^*^(H^+^) are the solvation free energies of the neutral solvent species and the proton, respectively. The experimental values for the first three terms on the right side of eq 1 are known for both solvents (see Table 1). The solution acidity, Δ*G*_sol_^(1)^, can then be obtained from the equilibrium condition of the thermodynamic cycle 1

2 $$\Delta G_{sol}^{\left(1\right)}=-\mathrm{RT}\;\ln\left(\frac{[S^{-}]\left[H^{+}\right]}{\left[SH\right]}\right)=-\mathrm{RT}\;\ln\left(\frac{K_{ap}}{\left[SH\right]}\right)$$

where *R* is the ideal gas constant, *T* is the temperature, *K*_ap_ = [S^–^]­[H^+^] is the autoprotolysis constant for this reaction, and [SH] is the concentration of the solvent. Therefore, the solution acidity depends only on the temperature, the concentration of the solvent, and its autoprotolysis constant. These values are well-known for both solvents (see Table 1). For this thermodynamic cycle, p*K*_a_^(1)^ can be obtained from the acidity constant definition, *K*_a_^(1)^ = [S^–^]­[H^+^]/[SH], leading to the following equation

3 $$pK_{a}^{\left(1\right)}=\frac{\Delta G_{sol}^{\left(1\right)}}{\mathrm{RT}\;\ln10}$$

**Table 1:** Summary of the Experimental Data Available for the Thermodynamic Properties Involved in the Protonation/Deprotonation Process of the Solvent Molecules in Water and Methanol Solutions Presented in Schemes 1 and 3 ^a^

experimental data	in aqueous solution	in methanol solution
other works		
solvent concentration, [SH]	55.5 mol L^–1^	24.5 mol L^–1^
autoprotolysis constant, *K*_ap_	10^–14^ ^3^	10^–16.7^ ^20,23^
p*K*_a_ = −log*K*_ap_ + log[SH]	15.7	18.1
gas phase acidity, Δ*G*_gas_^(1)^	385.64 ± 0.10 ^52^	377.93 ± 0.62 ^53^
gas phase basicity, Δ*G*_gas_^(3)^	159.64 ± 1.90 ^54^	175.06 ± 1.90 ^54^
Δ_sol_^*^(SH)	–6.32 ± 0.20 ^55^	–4.86 ± 0.20 ^55^
Δ_sol_^*^(H^+^)	–265.90 ± 0.10 ^56^	–263.50 ± 2.00 ^57^
Δ*G*_sol_^*^(S^–^)	–105.0;^43^ −104.5;^41^ −104.7;^38^ 104.5 ^44^	unknown
Δ*G*_sol_^*^(SH_2_^+^)	–110.20 ^43^	unknown
this work		
corrected constant, *K*_ap_^*^ = 10^–2δ^ *K*_ap_		10^–21.3^ {10^–16.7^}
solution acidity, Δ*G*_sol_^(1)^ [using eq 2]	21.46	30.93 {24.66}
solution basicity, Δ*G*_sol_^(3)^ [using eq 7]	–2.38	–1.89
p*K*_a_^(1)^ [using eq 3]	15.7	22.7 {18.1}
Δ*G*_sol_^*^(S^–^) [using eq 1]	–104.60 ± 0.25	–88.36 ± 2.10 {−94.63}
Δ*G*_sol_^*^(SH_2_^+^) [using eq 5]	–110.20 ± 1.91	–91.41 ± 2.76

a The values obtained from previous works are presented at the top. The values obtained in this work are derived from the corrected autoprotolysis constant *K*_ap_^*^ {or the original *K*_ap_ in parentheses} (see text). All of the free energy values are in kcal mol^–1^.

From the thermodynamic cycle 2, the Δ*G*_sol_^(2)^ can be obtained from the equilibrium condition as

4 $$\Delta G_{\mathrm{sol}}^{\left(2\right)}=-\mathrm{RT}\;\ln\left(\frac{[S^{-}]\left[SH_{2}^{+}\right]}{{\left[\mathrm{SH}\right]}^{2}}\right)=-\mathrm{RT}\;\ln\left(\frac{K_{\mathrm{ap}}}{{\left[\mathrm{SH}\right]}^{2}}\right)$$

where *K*_ap_ = [S^–^]­[SH_2_^+^] is the autoprotolysis constant for this reaction. Δ*G*_sol_^(2)^ also depends only on the temperature, the concentration of the solvent, and its autoprotolysis constant. The p*K*_a_^(2)^ value is then obtained through the relation

5 $$pK_{a}^{\left(2\right)}=\frac{\Delta G_{\mathrm{sol}}^{\left(2\right)}}{\mathrm{RT}\;\ln10}-\log\left[\mathrm{SH}\right]$$

The value for Δ*G*_sol_^*^(SH_2_^+^) can now be obtained from the thermodynamic cycle shown in Scheme 3, using the gas phase and solution basicities of the solvent and the corresponding solvation free energies of the species involved in the process

6 $$\Delta G_{\mathrm{sol}}^{*}\left(SH_{2}^{+}\right)=\Delta G_{\mathrm{gas}}^{\left(3\right)}+\Delta G_{\mathrm{sol}}^{*}\left(\mathrm{SH}\right)+\Delta G_{\mathrm{sol}}^{*}\left(H^{+}\right)-\Delta G_{\mathrm{sol}}^{\left(3\right)}$$

The experimental values for the first three terms on the right side of eq 6 are known for both solvents (see Table 1). The gas phase or solution basicity, Δ*G* ^(3)^, is related to those of Δ*G* ^(1)^ and Δ*G* ^(2)^. This relationship can be obtained assuming a two-step process in the thermodynamic cycle 2: $2\mathrm{SH}\overset{\Delta G^{\left(2\right)}}{\to }S^{-}+SH_{2}^{+}$ is equal to $2\mathrm{SH}\overset{\Delta G^{\left(1\right)}}{\to }S^{-}+H^{+}+\mathrm{SH}\overset{-\Delta G^{\left(3\right)}}{\to }S^{-}+SH_{2}^{+}$ . Therefore, Δ*G* ^(2)^ = Δ*G* ^(1)^ – Δ*G* ^(3)^, leading to the relation

7 $$\Delta G^{\left(3\right)}=\Delta G^{\left(1\right)}-\Delta G^{\left(2\right)}$$

Substituting Δ*G*_sol_^(1)^ (shown in eq 2) and Δ*G*_sol_^(2)^ (shown in eq 4) in eq 7, we obtain

8 $$\Delta G_{\mathrm{sol}}^{\left(3\right)}=-\mathrm{RT}\;\ln\left(\frac{K_{\mathrm{ap}}}{\left[\mathrm{SH}\right]}\right)+\mathrm{RT}\;\ln\left(\frac{K_{\mathrm{ap}}}{{\left[\mathrm{SH}\right]}^{2}}\right)=-\mathrm{RT}\;ln\left[\mathrm{SH}\right]$$

Thus, the solution basicity of solvent Δ*G*_sol_^(3)^ depends only on the temperature and the concentration of the solvent.

### 2.2 Results

The determination of Δ*G*_sol_^*^(S^–^) and Δ*G*_sol_^*^(SH_2_^+^) for both water and methanol solvents, using eqs 1 and 6, respectively, requires a priori knowledge of the solvation free energies of the proton, Δ*G*_sol_^*^(H^+^), of the neutral species, Δ*G*_sol_^*^(SH), in these solvents, as well as the solution acidity and basicity free energies for water and methanol.

The solution acidity, Δ*G*_sol_^(1)^, and the solution basicity, Δ*G*_sol_^(3)^, for both solvents can be determined using eqs 2 and 8, respectively, assuming *T* = 25 °C, *RT* = 0.5926 kcal mol^–1^, [H_2_O] = 55.5 mol L^–1^, [CH_3_OH] = 24.5 mol L^–1^, and the autoprotolysis constant, *K*_W_ = 10^–14^ for water and the correct value *K*_MOH_^*^ = 10^–2δ^ *K*_MOH_ = 10^–21.3^, with δ = 2.30 for methanol and the original value *K*_MOH_ = 10^–16.7^. These values are shown in Table 1, leading to an aqueous acidity of Δ*G*_sol_^(1)^ = Δ*G*_W_^(1)^ = 21.46 kcal mol^–1^ and an aqueous basicity of Δ*G*_sol_^(3)^ = Δ*G*_W_^(3)^ = −2.38 kcal mol^–1^, a methanol acidity of Δ*G*_sol_^(1)^ = Δ*G*_MOH_^(1)^ = 30.93 {24.66 for *K*_MOH_} kcal mol^–1^ and a methanol basicity of Δ*G*_sol_^(3)^ = Δ*G*_MOH_^(3)^ = −1.89 kcal mol^–1^. As we can see, there is a difference of 6.27 kcal mol^–1^ (=2δ*RT* ln 10) between both values for the methanol acidity Δ*G*_MOH_^(1)^ obtained with the corrected autoprotolysis constant, *K*_ap_^*^ = *K*_MOH_^*^ = 10^–2δ^ *K*_MOH_ = 10^–21.3^ and the original one {*K*_ap_ = *K*_MOH_ = 10^–16.7^}. The use of *K*_ap_^*^ {or *K*_ap_} will also reveal differences in the solvation free energy of the anionic species Δ*G*_sol_^*^(S^–^) and the p*K*_a_^(1)^ according the eqs 1 and 3, respectively.

The standard solvation free energies of the proton have been determined by several authors both in water^7,36,38,41,44,56,58–70^ and in methanol^57,71–73^ solution. Therefore, we have used the recommended values of the aqueous solvation free energy of the proton, Δ*G*_sol_^*^(H^+^) = Δ*G*_W_^*^(H^+^) = −265.90 ± 0.10 kcal mol^–1^, obtained by Tissandier et al.^56^ and the methanol solvation free energy of the proton, Δ*G*_sol_^*^(H^+^) = Δ*G*_MOH_^*^(H^+^) = −263.50 ± 2.00 kcal mol^–1^, obtained by Kelly et al.^57^ For the solvation free energies of water and methanol into their neat liquids, we have used the values of Δ*G*_sol_^*^(SH) = Δ*G*_W_^*^(H_2_O) = −6.32 ± 0.20 kcal mol^–1^ and Δ*G*_sol_^*^(SH) = Δ*G*_MOH_^*^(CH_3_OH) = −4.86 ± 0.20 kcal mol^–1^.^55^ In eq 1, we have used the experimental gas-phase acidity values recommended by the NIST tables,^74^ i.e., Δ*G*_gas_^(1)^ = 385.64 ± 0.10 kcal mol^–1^ for water, as reported by Smith et al.,^52^ and Δ*G*_gas_^(1)^ = 377.93 ± 0.62 kcal mol^–1^ for methanol, as reported by Nee et al.^53^ In eq 6, we have used the experimental gas-phase basicity values for water and methanol, namely, Δ*G*_gas_^(3)^ = 159.64 ± 1.90 and 175.06 ± 1.90 kcal mol^–1^, respectively.^54^ A summary of these values for Δ*G*_sol_^*^(H^+^), Δ*G*_sol_^*^(SH), Δ*G*_gas_^(1)^, and Δ*G*_gas_^(3)^ is presented at the top of Table 1 for aqueous and methanol solutions.

Table 1 summarizes all of the experimental data used to determine the values of Δ*G*_sol_^*^(S^–^) and Δ*G*_sol_^*^(SH_2_^+^) for both water and methanol solvents in the thermodynamic cycles shown in Schemes 1–3, and eqs 1 and 6, respectively. This approach leads to the values for the standard solvation free energies of the hydroxide in water, Δ*G*_sol_^*^(S^–^) = Δ*G*_W_^*^(OH^–^) = −104.60 ± 0.25 kcal mol^–1^ and the hydronium in water, Δ*G*_sol_^*^(SH_2_^+^) = Δ*G*_W_^*^(H_3_O^+^) = −110.20 ± 1.91 kcal mol^–1^, where the error bars were obtained using the propagation of the experimental errors. The values obtained in this work are in excellent agreement with the most reliable available experimental results for the solvation free energies of the hydronium in water, Δ*G*_W_^*^(H_3_O^+^), as −110.2 kcal mol^–1^ obtained by Pliego and Riveros,^43^ and for the hydroxide in water, Δ*G*_W_^*^(OH^–^), as (i) −105 kcal mol^–1^ obtained by Pliego and Riveros;^43^ (ii) −104.5 kcal mol^–1^ obtained by Palascak and Shields^36^ after correction by 1.9 kcal mol^–1^, as shown by Camaioni and Schwerdtfeger^41^; (iii) −104.7 kcal mol^–1^ obtained by Kelly et al.;^37,38^ and (iv) −104.5 kcal mol^–1^ reported by Zhan and Dixon.^44^ Although there is a general consensus about the value of Δ*G*_W_^*^(H_3_O^+^) = −110.20 kcal mol^–1^, there is a small uncertainty (about 0.5 kcal mol^–1^) with regard to the true value of Δ*G*_W_^*^(OH^–^) = −104.5 to −105.0 kcal mol^–1^. A similar procedure can be used to evaluate the values of Δ*G*_sol_^*^(S^–^) and Δ*G*_sol_^*^(SH_2_^+^) for other nonaqueous solvents. Using this approach for methanol, we obtain an experimental estimate for the solvation free energies of the methoxonium and methoxide ions in methanol, Δ*G*_MOH_^*^(CH_3_OH_2_^+^) = −91.41 ± 2.76 kcal mol^–1^ and Δ*G*_MOH_^*^(CH_3_O^–^) = −88.36 ± 2.10 {or −94.63 using the uncorrected *K*_ap_} kcal mol^–1^, respectively.

## 3 Computational Methods

In this work, we studied the individual neutral water and methanol molecules (H_2_O and CH_3_OH), their protonated species (H_3_O^+^ and CH_3_OH_2_^+^), and their respective deprotonated species (OH^–^ and CH_3_O^–^). The geometry of the different species was initially optimized using quantum mechanics (QM) calculations at the Møller–Plesset second order perturbation theory (MP2)^75,76^ level of theory with the basis set functions, 6–311++G­(d,p). After geometry optimization, the vibrational frequencies were calculated to determine the gas phase free energy of each molecule, *G*_gas_(*X*), using the electronic energies calculated at the fourth-order perturbation MP4 level and zero-point, enthalpy, and thermal corrections at the MP2 level with the same basis set.

The standard solvation free energy of each *X* species, Δ*G*_sol_^*^(*X*), in water and in methanol was calculated using the Free Energy Perturbation method (FEP)^77–80^ implemented in the Monte Carlo (MC) simulations, as used before,^81–86^ and named here as FEP-MC. For comparison purposes, we also calculated the Δ*G*_sol_^*^(*X*) by QM calculations, with the solvent described by three pure implicit solvent models: the polarizable continuum model (PCM),^87^ the Conductor-like Polarizable Continuum Model (C-PCM),^48^ and the solvation model based on density (SMD).^49^ For the PCM calculations, we used the Hartree–Fock (HF) method with 6–31+G­(d) Pople basis set functions,^88^ and a United Atom for Hartree–Fock (UAHF) model for the cavity with atomic radii optimized at the HF/6–31+G­(d) level of theory.^89^ This PCM/UAHF/HF/6–31+G­(d) method (named here as HF-PCM) has been successful in calculating the solvation free energy of neutral, cationic, and anionic species.^90–93^ For calculations using the C-PCM model, we employed the B3LYP/6–31G­(d,p) method, while for the SMD calculations, we employed the M062*X*/6–31+G­(d) approach.

Additionally, to increase the numerical precision of Δ*G*_sol_^*^(*X*), for *X* = SH_2_^+^ and S^–^, the SMD calculations were repeated, incorporating a few explicit solvent molecules alongside the implicit SMD solvation model. Specifically, three solvent molecules were explicitly incorporated into the ionic species, as previous studies have demonstrated that this approach improves the calculated solvation free energies compared to the original model.^94–97^ However, calculations involving clusters formed by an ion surrounded by three explicit solvent molecules incur in a higher computational cost, and the obtained values depend on the global minima of the clusters which are challenging to be determined.^50,98^ Thus, to address the challenge posed by the global minima of the cluster, we utilized 200 configurations obtained from MC simulations of both SH_2_^+^ and S^–^ interacting with three solvent molecules via hydrogen bonds (see Figure SM1 in the Supporting Information). Subsequently, we performed QM calculations using this hybrid model (i.e., implicit + explicit, named here as cluster-SMD) at the same level of QM calculation as the pure implicit SMD solvation model. For these calculations, solvent descriptors for water and methanol were obtained from the Minnesota Solvent Descriptor Database.^99^

The MC simulations were carried out with the Metropolis sampling technique and standard procedures, as previously illustrated.^100^ Six different systems were simulated. Each system consisted of one solute *X* and 500 solvent molecules in the isothermal–isobaric NPT ensemble, where the number of molecules *N* = 1 + 500, the pressure *P* = 1 atm, and the temperature *T* = 25 °C. The simulated systems were: H_2_O, H_3_O^+^, and OH^–^ in aqueous solution, and CH_3_OH, CH_3_OH_2_^+^, and CH_3_O^–^ in methanol solution. The periodic boundary conditions and the image method were used in a cubic box that was initialized with an experimental density of 1.000 g cm^–3^ for water and 0.781 g cm^–3^ for methanol. The geometry and the potential parameters of the molecules are kept fixed during the simulations. Each molecule interacts with all other molecules within a spherical region that is defined by a cutoff radius, *r*_c_ = *L*/2, where *L* is the lateral dimension of the simulation box. The long-range corrections of the potential are calculated beyond this cutoff distance as before.^100^ The intermolecular interaction was described by the Lennard-Jones (LJ) plus Coulomb potential, where each interacting site *i* has three parameters (ε_*i*_, σ_*i*_, and *q*_*i*_), with the combination rule of $\varepsilon_{\mathrm{ij}}=\sqrt{\varepsilon_{i}\varepsilon_{j}}$ and $\sigma_{\mathrm{ij}}=\sqrt{\sigma_{i}\sigma_{j}}$ . We employed the following force field: the SPC model^101^ for water, and the five sites OPLS model^102^ for methanol, with the set of LJ parameters {ε_*i*_ and σ_*i*_} of the OPLS^102^ for all other neutral solutes SH. For all charged solutes SH_2_^+^ and S^–^, we used the same set of LJ parameters of the neutral solutes. For all solutes (SH, SH_2_^+^, and S^–^), we used a set of polarized atomic charges {*q*_*i*_}_pol_ that were calculated with the CHELPG procedure to fit the electrostatic potential^103^ at the MP2/6–311++G­(d,p) level of QM calculation, with the solute embedded in the solvent described by PCM. Therefore, the set of atomic charges used in the simulation for the solute molecules includes implicitly the electronic polarization due to the presence of the solvent. This procedure for generating the atomic charges for the solute coupled with the OPLS LJ parameters was used before.^86,104–106^ It has been shown to be better for describing the solvent effects on electronic properties than the standard procedure of calculating the set of atomic charges with HF/6–31G­(d), which includes an average polarization of 30% independent of the specific solution. Our results in this work show that this procedure is also good to describe the solvation free energy of simple molecules in water and methanol solutions. The potential parameters (Lennard–Jones {ε and σ} and the atomic charges {q}) of H_2_O, H_3_O^+^, OH^–^, CH_3_OH, CH_3_OH_2_^+^, and CH_3_O^–^ used in this work are shown in the Supporting Information.

The procedure used here to calculate the solvation free energy of each species, Δ*G*_sol_^*^(*X*), was obtained as the negative value of the annihilation free energy in solution, as previously illustrated.^81^ For each species, a series of several FEP-MC simulations were performed to make the vanishing process of the solute *X* divided into two stages: one to annihilate the electrostatic potential, −Δ*G*_ele_(*X*), i.e., the Coulomb potential, and the other to annihilate the nonelectrostatic interactions, −Δ*G*_nonele_(*X*), i.e., the LJ potential. The total value of the solvation free energy of each species was then obtained by adding these two different terms calculated with the FEP-MC simulation,^81,83,84,107^ with the term due to the changes of the ideal gas at standard concentration of 1 M to a condition of 1 atm in equilibrium with the solution

9 $$\Delta G_{\mathrm{sol}}^{*}\left(X\right)=-\Delta G_{\mathrm{ele}}\left(X\right)-\Delta G_{\mathrm{nonele}}\left(X\right)-\mathrm{RT}\;\ln\left(24.46\right)$$

The total annihilation of each solute *X* was performed in 20 simulations: (i) 12 simulations with double-wide sampling to annihilate the atomic charges λ_*i*_{*q*}_pol_, with λ_*i*_ = 1.000, 0.975, 0.950, 0.925, 0.900, 0.875, 0.850, 0.825, 0.800, 0.775, 0.750, 0.725, 0.700, 0.675, 0.650, 0.625, 0.600, 0.550, 0.500, 0.450, 0.400, 0.350, 0.300, 0.200, and 0.00, where, for each underlined λ_*i*_, a simulation was performed with double-wide sampling, i.e., λ_*i*–1_ ← λ*_i_* → λ_*i*+1_; and (ii) 8 simulations to annihilate the ones with λ_*i*_ = 1.000, 0.875, 0.750, 0.625, 0.500, 0.375, 0.250, 0.125, and 0.00, where 4 simulations were carried out with double-wide sampling to annihilate the attractive term of the LJ potential and 4 simulations without double-wide sampling to annihilate the repulsive term of the LJ potential with λ_*i*_ = 1.00, 0.75, 0.50, 0.25, and 0.00. For each simulation, five independent runs, with thermalization and equilibration both with 1.5 × 10^8^ MC steps, were performed to calculate the average and standard deviation of the free energy between the states. After the vanishing simulation process, the Δ*G*_sol_^*^(*X*) was corrected, considering: (i) the polarization free energy, due to the polarization of the solute in solution, and (ii) the standard reference state of 1 mol L^–1^. More details about this procedure can be found in ref 81.

Finally, we used the thermodynamic cycles of the acidity reaction (Scheme 1) and of the acid–base reaction (Scheme 2), and the calculated values of *G*_gas_(SH), *G*_gas_(H^+^), *G*_gas_(SH_2_^+^), and *G*_gas_(S^–^) obtained with QM calculations and Δ*G*_sol_^*^(SH), Δ*G*_sol_^*^(SH_2_^+^), and Δ*G*_sol_^*^(S^–^) obtained with the various solvation models FEP-MC, HF-PCM, C-PCM, SMD, and cluster-SMD, in both solvents, to calculate the solution acidity Δ*G*_sol_^(1)^ (using eq 2), Δ*G*_sol_^(2)^ (using eq 4), p*K*_a_^(1)^ (using eq 3), and p*K*_a_^(2)^ (using eq 5). All of the QM calculations were performed with the Gaussian 03 program,^108^ except for calculations employing the C-PCM and SMD solvation models, which were performed using Gaussian 16.^109^ All FEP-MC simulations were performed using the DICE program.^110^

## 4 Results from Theoretical Calculations and Discussion

### 4.1 Chemical Equilibrium in the Gas Phase

The free energy of each species in vacuum was calculated as described in Section 3, and the results are shown in Table 2.

**Table 2:** Calculated Gas Phase Free Energies (in kcal mol^–1^) for the Species Involved in the Heterolytic Dissociation of Water and Methanol (See Scheme 2)­^a^

free energy in the gas phase	water	methanol
*G*_gas_(SH)	–47857.37	–72444.89
*G*_gas_(SH_2_^+^)	–48022.66	–72626.01
*G*_gas_(S^–^)	–47465.69	–72061.05
Δ*G*_gas_^(2)^	226.4	202.7
experimental Δ*G*_gas_^(2)^	226.0 ± 1.9 ^54^	202.9 ± 2.0 ^54^
experimental Δ*G*_gas_^(1)^	385.64 ± 0.10 ^52^	377.93 ± 0.60 ^53^
*G*_gas_(H^+^) = Δ*G*_gas_^(1)^ + *G*_gas_(SH) – *G*_gas_(S^–^)	–6.04 ± 0.10	–5.91 ± 0.60

a The geometries were optimized at the MP2/6-311++G­(d,p) level of quantum mechanics calculation, and the appropriate zero-point, thermal, and enthalpy corrections of the energy were obtained after the vibrational frequencies calculations. The electronic energies were calculated at the MP4 level using the same basis function.

The gas phase free energies, Δ*G*_gas_^(2)^, for the process 2SH → S^–^ + SH_2_^+^ and the gas phase acidities, Δ*G*_gas_^(1)^, (SH → S^–^ + H^+^) were calculated for water and methanol using the free energy of the involved species. The calculated values are displayed in Table 2 and compared with the experimental values.

As we can see from Table 2, our calculations yield Δ*G*_g_^(2)^(water) = 226.4 kcal mol^–1^ and Δ*G*_g_^(2)^(methanol) = 202.7 kcal mol^–1^, in excellent agreement with the experimental values of Δ*G*_g_^(2)^(water) = 226.0 kcal mol^–1^ and Δ*G*_g_^(2)^(methanol) = 202.9 kcal mol^–1^, as determined from eq 7, where the values used for Δ*G*_g_^(3)^ and Δ*G*_g_^(1)^ are listed in the Table 1. A value of −6.04 kcal mol^–1^ was obtained for *G*_g_(H^+^) for water using the experimental values of Δ*G*_g_^(1)^(H_2_O → OH^–^ + H^+^) = 385.64 kcal mol^–1^, along with the calculated values of *G*_g_(H_2_O) and the *G*_g_(OH^–^). For methanol, a value of −5.91 kcal mol^–1^ was obtained for *G*_g_(H^+^) from the experimental values of Δ*G*_g_^(1)^(CH_3_OH → CH_3_O^–^ + H^+^) = 377.93 kcal mol^–1^, together with the calculated values of *G*_g_(CH_3_OH) and the *G*_g_(CH_3_O^–^). For further calculations, we have adopted an average value of *G*_g_(H^+^) = −6.0 kcal mol^–1^, which is in excellent agreement with the value of −6.28 kcal mol^–1^ obtained from the Sackur–Tetrode equation.^111^

### 4.2 Chemical Equilibrium in the Solution

The values of Δ*G*_gas_^(1)^, Δ*G*_gas_^(2)^, Δ*G*_sol_^*^(SH), Δ*G*_sol_^*^(H^+^), Δ*G*_sol_^*^(SH_2_^+^), and Δ*G*_sol_^*^(S^–^) are of fundamental importance for studying the processes Δ*G*_sol_^(1)^(SH → S^–^ + H^+^) and Δ*G*_sol_^(2)^(2SH → S^–^ + SH_2_^+^) of solvent molecules in their own liquids, as these properties are directly correlated to the acid dissociation constants (see eqs 3 and 5).

The values of Δ*G*_sol_^*^(*X*) were obtained from eq 9, while the values of Δ*G*_sol_^(1)^ were obtained from Scheme 1 and eq 2, and Δ*G*_sol_^(2)^ were obtained from Scheme 2 and eq 4. Finally, the p*K*_a_ of each solute molecule in each scheme was obtained using eq 3 (p*K*_a_^(1)^) and eq 5 (p*K*_a_^(2)^). The main results are summarized in Table 3.

**Table 3:** Standard Solvation Free Energies (in kcal mol^–1^) of Solvent Molecules Involved in the Acidity Reaction (Scheme 1) and Acid–Base Reaction (Scheme 2) in Water and Methanol^a^

methods	Δ*G*_sol_(SH)^b^	Δ*G*_sol_(S^–^)^c^	Δ*G*_sol_(SH_2_^+^)	Δ*G*_sol_^(1)^ ^d^	Δ*G*_sol_^(2)^ ^e^	p*K*_a_^(1)^	p*K*_a_^(2)^
Water in Aqueous Solution							
FEP-MC	–6.6 ± 0.9	–105.5 ± 0.7	–94.3 ± 1.0	20.8 ± 1.5	39.8 ± 2.4	15.3 ± 1.1	27.4 ± 1.8
HF-PCM	–7.5	–106.0	–107.6	21.2	27.8	15.5	18.6
C-PCM	–7.0	–95.4	–90.7	31.3	54.3	23.0	38.1
SMD	–6.4	–92.8	–96.1	33.3	50.3	24.4	35.1
cluster-SMD^f^	–6.4	–102.46 ± 1.72	–110.50 ± 0.20	23.68 ± 1.74	26.24 ± 2.56	17.36 ± 1.27	17.49 ± 1.88
experimental	–6.32 ± 0.20	–104.60 ± 0.20	–110.20 ± 1.91	21.46 ± 1.94	23.84 ± 2.00	15.73 ± 1.42	15.73 ± 1.46
Methanol in Methanol Solution							
FEP-MC	–5.6 ± 0.2	–87.1 ± 0.6	–74.9 ± 0.3	32.9 ± 2.2	51.9 ± 2.1	24.1 ± 1.6	36.7 ± 1.5
HF-PCM	–6.9	–86.5	–90.4	34.8	39.6	25.5	27.6
C-PCM	–4.9	–74.2	–75.9	45.1	62.4	33.1	44.4
SMD	–6.3	–81.4	–81.8	39.3	52.1	28.8	36.8
cluster-SMD^f^	–6.3	–89.95 ± 0.50	–91.36 ± 0.45	30.78 ± 2.20	33.99 ± 2.06	22.56 ± 1.61	23.53 ± 1.51
experimental	–4.86 ± 0.20	–88.36 ± 2.10	–91.41 ± 2.76	30.93 ± 4.00	32.85 ± 3.00	22.67 ± 2.97	22.69 ± 2.20

a The values were calculated using FEP-MC simulation, a hybrid model (cluster-SMD), and three continuum solvation models: HF-PCM, C-PCM, and SMD. Additionally, experimental data is provided for comparison.

b Experimental values of Δ*G*_sol_(SH) in water obtained from ref 55.

c Experimental values obtained from Scheme 1, using the combination of eqs 1 and 3, with values of Δ*G*_gas_^(1)^, showed in Table 2.

d Experimental values obtained from Scheme 1.

e Experimental values obtained from Scheme 2.

f Values for anionic and cationic species were calculated using the cluster-SMD model, whereas neutral species were calculated using the pure implicit SMD.

The results for the standard solvation free energies, Δ*G*_sol_^*^(*X*), of the neutral forms *X* = H_2_O and CH_3_OH in water and methanol are −6.6 ± 0.9 and −5.6 ± 0.2 kcal mol^–1^, respectively, obtained using the FEP-MC model. In comparison with the pure implicit solvation models (HF-PCM, C-PCM, and SMD), which produce solvation free energies of (−7.5, −7.0, and −6.4 kcal mol^–1^) for water and (−6.9, −4.9, and −6.3 kcal mol^–1^) for methanol, respectively, we observe excellent agreement between both explicit and implicit solvation models. Specifically, the FEP-MC model, which explicitly includes solvent molecules in the calculation, agrees well with the HF-PCM, C-PCM, and SMD models, which consider the solvent as a continuum medium. When comparing these results to the experimental values of Δ*G*_sol_^*^(H_2_O) = −6.32 kcal mol^–1^ and Δ*G*_sol_^*^(CH_3_OH) = −4.86 kcal mol^–1^, as reported by Ben-Naim and Marcus,^55^ we observe excellent agreement between the calculated and the experimental results. The SMD model yields the best result for water, while the C-PCM model provides the best result for methanol.

For cations H_3_O^+^ and CH_3_OH_2_^+^, the FEP-MC, HF-PCM, C-PCM, and SMD models yield standard solvation free energies of −94.3 ± 1.0, −107.6, −90.7, and −96.1 kcal mol^–1^ for water and −74.9 ± 0.3, −90.4, −75.9, and −81.8 kcal mol^–1^ for methanol, respectively. As can be seen from Table 3, these models predict standard solvation free energies for cations in relatively poor agreement with the experiment values (Δ*G*_sol_^*^(H_3_O^+^) = −110.20 ± 1.91 kcal mol^–1^ and Δ*G*_sol_^*^(CH_3_OH_2_^+^) = −91.41 ± 2.76 kcal mol^–1^). The most accurate solvation model among those that have been used for the cations is the HF-PCM model, which gives a difference between the calculated and experimental values of 2.6 kcal mol^–1^ for water and 1.0 kcal mol^–1^ for methanol.

To improve the accuracy of the calculated solvation free energies of ions in water and methanol, studies with the SMD solvation model were repeated by incorporating three explicit solvent molecules in this model, referred to above as the cluster-SMD. Figure 2 shows the calculated averages for Δ*G*_sol_^*^(H_3_O^+^) and Δ*G*_sol_^*^(CH_3_OH_2_^+^) obtained using the cluster-SMD for 200 MC configurations. As depicted in the inset images (Figure 2a,b), these properties converge after approximately 100 configurations for both water and methanol. We obtained converged average values for the standard solvation free energies of −110.50 ± 0.20 kcal mol^–1^ for the hydronium ion in water, and −91.36 ± 0.45 kcal mol^–1^ for the methoxonium ion in methanol. These results show that the cluster-SMD model provides a significant improvement compared with the original model (SMD) and with the other solvation models utilized in this study. These results exhibit excellent agreement with the experimental values, with a difference of less than 0.5 kcal mol^–1^.

**Figure 2:**
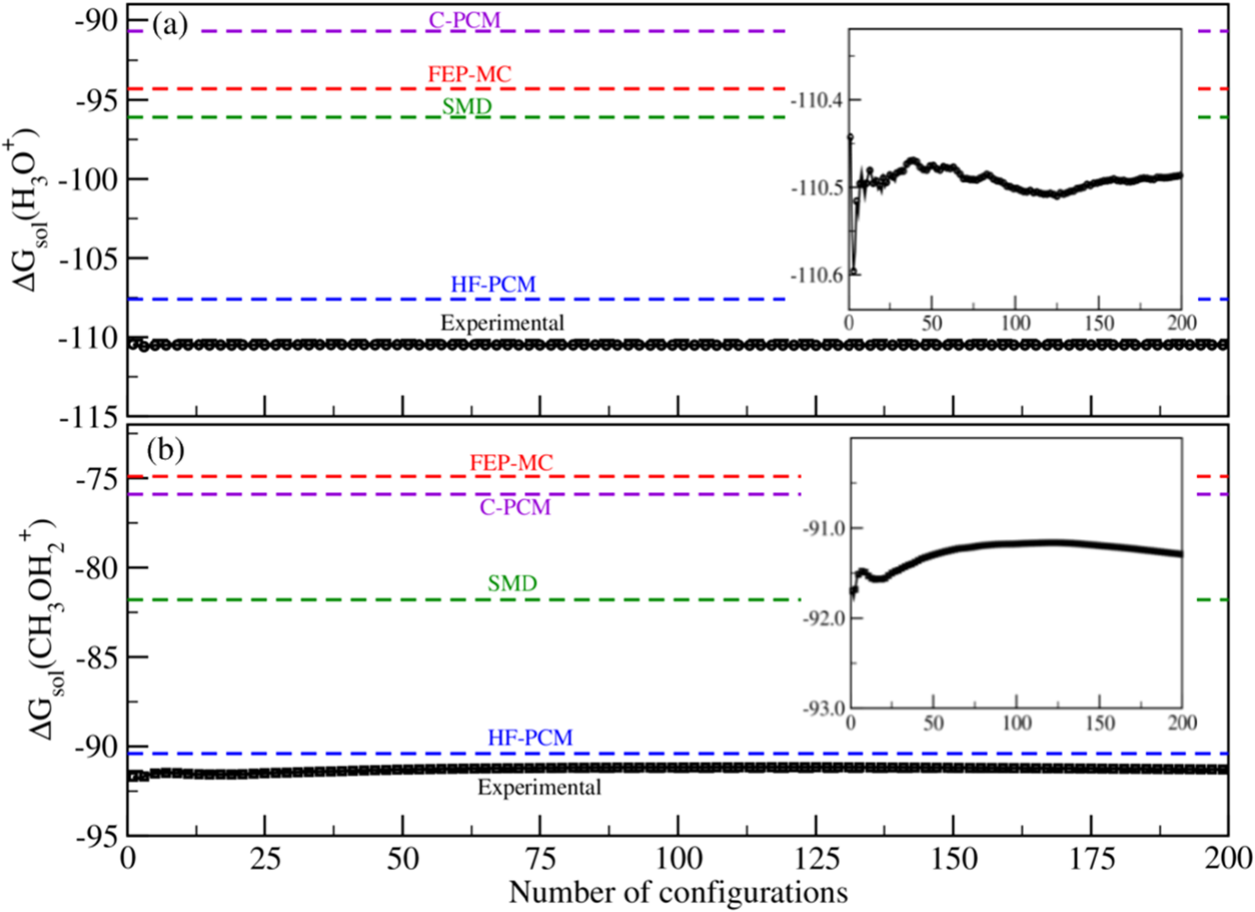
Convergence of the average value of the solvation free energy for cations, hydronium, and methoxonium in water (a) and methanol (b), respectively, as obtained by using the cluster-SMD model. The horizontal dashed lines show the experimental and theoretical values of Δ*G*_sol_^*^ obtained using the solvation models FEP-MC, HF-PCM, C-PCM, and SMD. The inset image shows a magnified view of the data along the *y*-axis.

Figure 3 shows the calculated average for the solvation free energies for the hydroxide and methoxide anions in water and methanol obtained using the cluster-SMD. Analysis of the Δ*G*_sol_^*^(OH^–^) and Δ*G*_sol_^*^(CH_3_O^–^) curves indicates that these properties converge after approximately 75 configurations for both anionic species (see insert images in Figure 3a,b). This analysis gives a converged average value of – 102.46 ± 1.72 kcal mol^–1^ for the standard solvation free energy of hydroxide in water and −89.95 ± 0.50 kcal mol^–1^ for methoxide in methanol. By comparison with the values obtained by the FEP-MC, HF-PCM, C-PCM, and SMD models of −105.5 ± 0.7, −106.0, −95.4, and −92.8 kcal mol^–1^ for OH^–^ and −87.1 ± 0.6, −86.5, −74.2, and −81.4 kcal mol^–1^ for CH_3_O^–^, the calculated values using cluster-SMD are in reasonable agreement with those values calculated using FEP-MC and HF-PCM models. The calculated values using C-PCM and SMD differ from the cluster-SMD by 7.1 and 9.7 kcal mol^–1^ for hydroxide and by 15.8 and 8.6 kcal mol^–1^ for methoxide. This large discrepancy is mainly attributed to the weak polarization effects of the anions, which systematically are undersolvated by 7–16 kcal mol^–1^ in the SMD and C-PCM models when compared with the cluster-SMD model. Although the calculations become computationally very expensive when three solvent molecules are explicitly considered, the cluster model convincingly demonstrates to be a robust approach for accurate estimates of the solvation free energy of anionic and cationic species in water and methanol.

**Figure 3:**
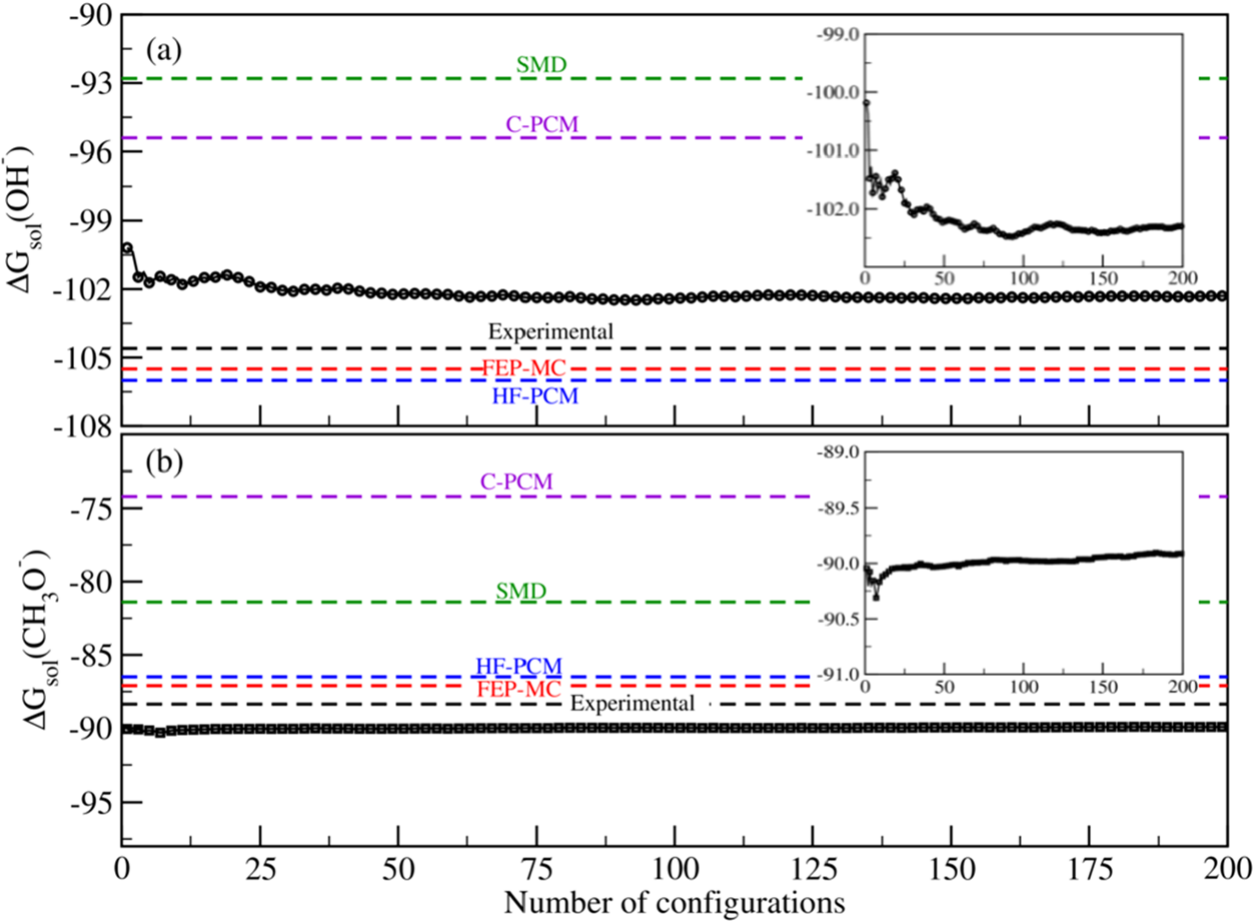
Convergence of the average value of solvation free energy for the anions, hydroxide, and methoxide in water (a) and methanol (b), respectively, as obtained by using the cluster-SMD model. The horizontal dashed lines show the experimental and theoretical values of Δ*G*_sol_^*^ obtained using the solvation models FEP-MC, HF-PCM, C-PCM, and SMD. The inset image shows a magnified view of the data along the *y*-axis.

The theoretical results obtained with FEP-MC, HF-PCM, and cluster-SMD for the hydroxide ion exhibit reasonable agreement with the experimental value of Δ*G*_sol_^*^(OH^–^) = −104.60 kcal mol^–1^ obtained in this study. For these solvation models, the differences between the theoretical and experimental values of Δ*G*_sol_^*^(OH^–^) are 0.9, 1.4, and 2.1 kcal mol^–1^, respectively. For the other implicit solvation models, C-PCM and SMD, these values are larger (9.2 and 11.8 kcal mol^–1^, respectively). Therefore, considering the theoretical results for hydroxide, the solvation models FEP-MC, HF-PCM, and cluster-SMD provide the best results with an error of approximately 1–2 kcal mol^–1^.

Comparing the theoretical results obtained with FEP-MC, HF-PCM, and cluster-SMD with the experimental value of −88.36 {−94.63} kcal mol^–1^ for methoxide, as shown in Table 1, we find a difference between the theoretical and experimental results of 1.3 {7.5} kcal mol^–1^ for FEP-MC, 1.9 {8.1} kcal mol^–1^ for HF-PCM, and 1.6 {4.7} kcal mol^–1^ for the cluster-SMD model, with the experimental values associated with the *K*_MOH_^*^ and {*K*_MOH_} constants. It is worth noting that the three solvation models achieve errors of 1–2 kcal mol^–1^ in the solvation free energy of CH_3_O^–^ in methanol, yielding errors similar in magnitude to those obtained in the studies of the hydroxide ion in aqueous solution. These results lead us to conclude that the experimental value of −88.36 kcal mol^–1^ associated with *K*_MOH_^*^ = 10^–21.3^ is our best experimental estimate for the solvation free energy of the methoxide ion in methanol. Hence, it is crucial to adjust the original value of the autoprotolysis constant to achieve thermodynamic significance for the solvation free energy of the methoxide ion in a methanol solution. This value of Δ*G*_sol_^*^(CH_3_O^–^) can now be used alongside the values of Δ*G*_sol_^*^(H^+^) = −263.5 kcal mol^–1^, Δ*G*_sol_^*^(CH_3_OH_2_^+^) = −91.41 ± 2.76 kcal mol^–1^, and Δ*G*_sol_^*^(CH_3_OH) = −4.89 kcal mol^–1^ for a consistent description of the Δ*G*_sol_^(1)^(CH_3_OH → CH_3_O^–^ + H^+^) and Δ*G*_sol_^(2)^(2CH_3_OH → CH_3_O^–^ + CH_3_OH_2_^+^) processes of the methanol molecule in its own liquid.

The solvation effects of the anionic and cationic species on the p*K*_a_ values were also explored using the various solvation models. We used thermodynamic cycles 1 and 2 to calculate the standard deprotonation free energies of the solvent molecules in their own liquids by direct dissociation, Δ*G*_sol_^(1)^ (Scheme 1), and by acid–base reaction, Δ*G*_sol_^(2)^(Scheme 2), along with their respective p*K*_a_^(1)^ and p*K*_a_^(2)^ values. Using Scheme 1, we obtained Δ*G*_W_^(1)^ as 20.8 ± 1.5 (21.2, 31.3, 33.3, and 23.68 ± 1.74) kcal mol^–1^ and Δ*G*_MOH_^(1)^ as 32.9 ± 2.2 (34.8, 45.1, 39.3, and 30.78 ± 2.20) kcal mol^–1^ with the FEP-MC (HF-PCM, C-PCM, SMD, and cluster-SMD) solvation models. Similarly, using Scheme 2, we obtained Δ*G*_W_^(2)^ as 39.8 ± 2.4 (27.8, 54.3, 50.3, and 26.24 ± 2.56) kcal mol^–1^ and Δ*G*_MOH_^(2)^ as 51.9 ± 2.1 (39.6, 62.4, 52.1, and 33.99 ± 2.06) kcal mol^–1^, respectively. Note that the differences in Δ*G*_sol_^(1)^ between FEP-MC and (HF-PCM and cluster-SMD) solvation models are relatively small (0.4 and 2.9 kcal mol^–1^ for water, and 1.9 and 2.1 kcal mol^–1^ for methanol), compared to the differences between FEP-MC and (C-PCM and SMD). The satisfactory agreement in values obtained with FEP-MC, HF-PCM, and cluster-SMD is attributed to the cancellation of the nonelectrostatic term for SH and S^–^ that are very similar. In contrast, significantly larger discrepancies are observed in Δ*G*_sol_^(2)^ between FEP-MC and the various solvation models (HF-PCM, C-PCM, SMD, and cluster-SMD), except for the result obtained with SMD for methanol, where the difference is notably smaller. These discrepancies can be attributed to inaccuracies in describing the electrostatic contributions of cationic species through these solvation models.

Finally, these solvation models provide different calculated p*K*_a_^(1)^ values for water in aqueous solution, 15.3 ± 1.1 (15.5, 23.0, 24.4, and 17.36 ± 1.27), and for methanol in methanol solution 24.1 ± 1.6 (25.5, 33.1, 28.8, and 22.56 ± 1.61), using Scheme 1, with FEP-MC (HF-PCM, C-PCM, SMD, and cluster-SMD), respectively. Similarly, the same methods yield p*K*_a_^(2)^ values for water in aqueous solution, 27.4 ± 1.8 (18.6, 38.1, 35.1, and 17.49 ± 1.88), and for methanol in methanol solution, 36.7 ± 1.5 (27.6, 44.4, 36.8, 23.53 ± 1.51) using Scheme 2, respectively. As can be seen from Table 3, the FEP-MC, HF-PCM, and cluster-SMD solvation models yield calculated p*K*_a_^(1)^ values in good agreement with experimental values (p*K*_a_(water) = 15.7 ± 0.2 and p*K*_a_(methanol) = 22.7 ± 2.2), with errors of less than 2 units of p*K*_a_^(1)^ for water and 3 units of p*K*_a_^(1)^ for methanol. However, for the p*K*_a_^(2)^ (see Table 3), the FEP-MC exhibited errors with more than 10 units of p*K*_a_^(2)^, whereas errors for HF-PCM and cluster-SMD are less than 3 units of p*K*_a_^(2)^ for water and 5 units of p*K*_a_^(2)^ for methanol. Therefore, FEP-MC, HF-PCM, and cluster-SMD solvation models showed lower errors and, consequently, better agreement with the experimental data for water and methanol in their respective liquids when used in Scheme 1. For Scheme 2, the HF-PCM and cluster-SMD models exhibit the best agreement.

It is noteworthy that the inaccuracies in the calculated p*K*_a_ values obtained using the FEP-MC model in Scheme 2, compared to the solvation models (HF-PCM and cluster-SMD), stem from errors in the calculation of the solvation free energies of the cations, as discussed above. Conversely, the significant errors observed with C-PCM and SMD, in comparison to the solvation models (HF-PCM and cluster-SMD), result from the lower precision of the calculated solvation free energies of both anionic and cationic species by these two implicit solvation models, as reported in previous studies.^49,112–115^

## 5 Conclusions

In this paper, we have shown that a correction for the autoprotolysis constant of methanol (*K*_MOH_^*^ = 10^–2δ^ *K*_MOH_) is necessary to obtain significant thermochemical quantities. This corrected constant, *K*_MOH_^*^, together with the most accurate values for the solvation free energies of a proton and neutral methanol in neat methanol, along with the gas phase basicity and acidity of methanol, lead to experimental solvation free energy values of −91.41 kcal mol^–1^ for the methoxonium ion and −88.36 kcal mol^–1^ for the methoxide ion in pure methanol. By comparison, a similar procedure using the well-established value for water, *K*_W_, along with solvation free energies of a proton in water and the gas phase basicity and acidity of water, leads to solvation free energies of hydronium and hydroxide ions in water, which are −110.20 and −104.60 kcal mol^–1^, respectively, in excellent agreement with previous results. Therefore, we conclude that correction of the original constant due to the difference between the pH* and pH_app_ scale in methanol is required to provide a full description of the thermodynamics of dilute methanol solutions.

Additionally, we combined quantum mechanics calculations, along with Monte Carlo simulations, to calculate the p*K*_a_ of water and methanol in their respective liquids. For Schemes 1 and 2, the calculated gas phase acidity, Δ*G*_gas_^(1)^, gas phase of heterolytic dissociation, Δ*G*_gas_^(2)^, and standard solvation free energies of water (H_2_O) and its conjugate base (OH^–^) in aqueous solution, of methanol (CH_3_OH) and its conjugate base (CH_3_O^–^) in methanol solution, agree well with experimental data. Considering Schemes 1 and 2, we calculated the standard deprotonation free energy of water and methanol in their respective liquids by two different thermodynamic cycles using different solvation models (FEP-MC, HF-PCM, C-PCM, SMD, and cluster-SMD). The best results are achieved using the HF-PCM (Δ*G*_W_ = 21.2 kcal mol^–1^ and Δ*G*_MOH_ = 34.8 kcal mol^–1^) and cluster-SMD (Δ*G*_W_ = 23.68 ± 1.74 kcal mol^–1^ and Δ*G*_MOH_ = 30.78 ± 2.20 kcal mol^–1^) methods. These two solvation models gave theoretical results in excellent agreement with the experimental values obtained in this study (Δ*G*_W_ = 21.46 ± 1.94 kcal mol^–1^ and Δ*G*_MOH_ = 30.93 ± 4.00 kcal mol^–1^), related to the acidity constants (p*K*_a_(water) = 15.73 ± 1.42 and p*K*_a_(methanol) = 22.67 ± 2.97), using eq 3.

It is noteworthy that for methanol, the most accurate results for the standard solvation free energies of methoxonium (−91.36 ± 0.45 (−90.4) kcal mol^–1^) and methoxide (−89.95 ± 0.50 (−86.5) kcal mol^–1^) ions in methanol were obtained using the cluster-SMD (HF-PCM) method. These results are consistent with studies using other solvation models (FEP-MC, C-PCM, and SMD) and also show better agreement with experimental data obtained from the corrected autoprotolysis constant, *K*_MOH_^*^, than with the original constant, *K*_MOH_. Therefore, we conclude that the correction of the original constant due to the difference between the pH* and pH_app_ scale in methanol is required to provide a full description of the thermodynamics of dilute methanol solutions. Moreover, these values can serve as benchmarks for reparameterization of many continuum solvation models that are currently parametrized to reproduce experimental aqueous solvation free energies. Our present results can therefore be used to evaluate the performance of various theoretical solvation models in reproducing solvation free energies of different ions in methanol.

## Supplementary Material



## Notes

Typical hydrogen-bonded structures used in the QM calculations using the cluster-SMD mode (Figure SM1), the Lennard-Jones plus Coulomb potential parameters ({ε} in kcal/mol, {σ} in Å, and {q} in e) used in the MC simulation of H_3_O^+^ and OH^–^ in aqueous solution, as well as CH_3_OH_2_^+^ and CH_3_O^–^ in methanol (Table SM1) (PDF)
