# Supplementary material for: Defining the pH* Scale in Methanol: Determination of Accurate Values for the Solvation Free Energies of CH3OH2 + and CH3O– in Methanol
Source: J Phys Chem A. 2025 Oct 25;129(44):10068–80. doi: 10.1021/acs.jpca.5c03979 (PMC12598864; doi:10.1021/acs.jpca.5c03979)
Supplement: Supplementary file 1 [file jp5c03979_si_001.pdf]

Supplementary Material

Defining the *pH** scale in methanol: Determination of accurate values for the solvation free energies of $CH_3OH_2^+$ and CH_3O^- in methanol.

Antonio R. Cunha*[1,3], José M. Riveros[2], Sylvio Canuto[3] and Kaline Coutinho[3].

[1]*Universidade Federal do Maranhão, UFMA, Campus Balsas, CEP 65800-000, Maranhão, Brazil.*

[2]*Instituto de Química, Universidade de São Paulo, CEP 05508-000, Cidade Universitária, São Paulo Brazil.*

[3]*Instituto de Física, Universidade de São Paulo, CP 05508-090, Cidade Universitária, São Paulo, Brazil.*

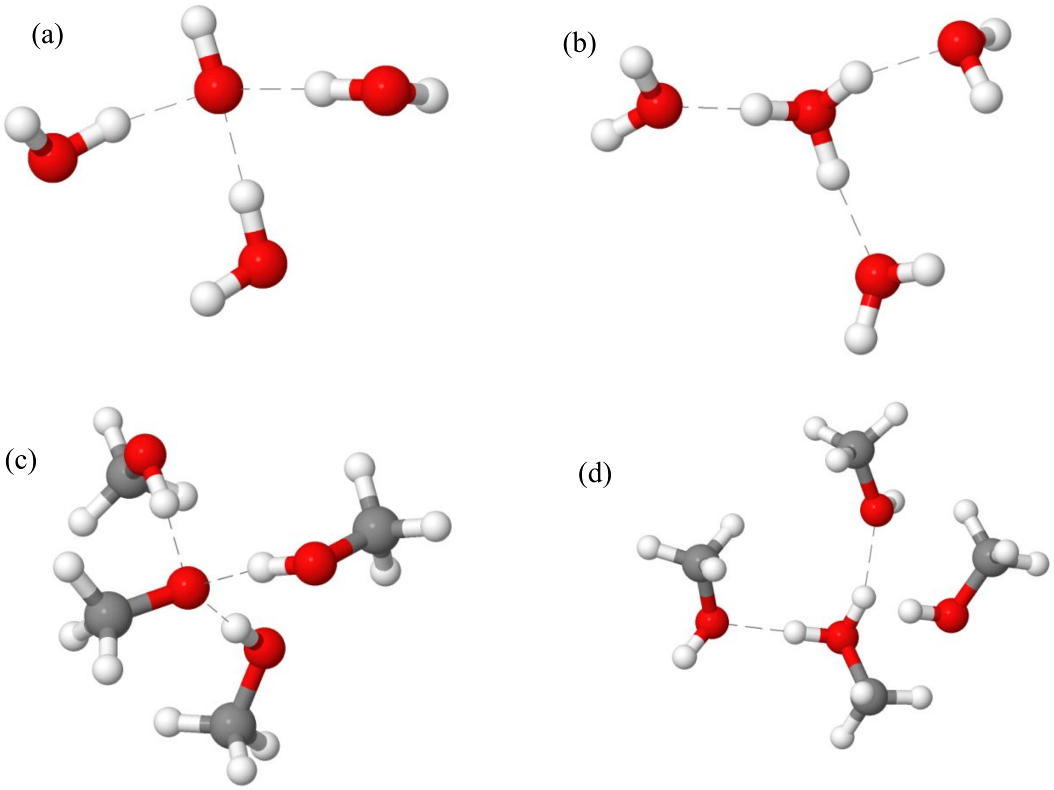

Figure SM1: Typical hydrogen-bonded structures used in the QM calculations using the cluster-SMD model. This illustration shows the (a) OH^-, (b) H_3O^+, (c) CH_3O^-, and (d) $CH_3OH_2^+$ surrounded by three-neighbor solvent molecules corresponding to the micro-solvation shell.

* Corresponding author: cunha.antonio@ufma.br

Table SM1: The Lennard-Jones plus Coulomb potential parameters ({ε} in kcal/mol, {σ} in Å, and {q} in e) used in the MC simulation of H_3O^+ and OH^- in aqueous solution, as well as $CH_3OH_2^+$ and CH_3O^- in methanol. The Lennard-Jones parameters were obtained from the SPC model [101] for water and from the OPLS force field [102] for methanol, while the atomic charges for the ion species were calculated using quantum mechanics with M062X/3-31+(d)/CHELPG/SMD for water and methanol in their respective liquids.

Atoms i	ε_i	σ_i	q_i
H_2O			
O1	0.155	3.165	-0.973
H1	0.000	0.000	0.487
H2	0.000	0.000	0.487
H_3O^+			
O1	0.155	3.165	-0.622
H1	0.000	0.000	0.540
H2	0.000	0.000	0.541
H3	0.000	0.000	0.541
OH^-			
O1	0.155	3.165	-1.429
H1	0.000	0.000	0.429
CH_3OH			
O1	0.170	3.120	-0.822
H1	0.000	0.000	0.498
C1	0.066	3.500	0.337
H2	0.030	2.500	0.025
H3	0.030	2.500	-0.019
H4	0.030	2.500	-0.019
$CH_3OH_2^+$			
O1	0.170	3.120	-0.504
H1	0.000	0.000	0.542
H2	0.000	0.000	0.542
C1	0.066	3.500	0.047
H3	0.030	2.500	0.101
H4	0.030	2.500	0.136
H5	0.030	2.500	0.136
CH_3O^-			
O1	0.170	3.120	-1.275
C1	0.066	3.500	0.752
H2	0.030	2.500	-0.158
H3	0.030	2.500	-0.159
H4	0.030	2.500	-0.159
